# Albuminuria confers renal resistance to loop diuretics via the stimulation of NLRP3 inflammasome/prostaglandin signaling in thick ascending limb

**DOI:** 10.18632/oncotarget.10257

**Published:** 2016-06-23

**Authors:** Yibo Zhuang, Zhanjun Jia, Caiyu Hu, Guixia Ding, Xintong Zhang, Yue Zhang, Guangrui Yang, Rajeev Rohatgi, Songming Huang, John Ci-Jiang He, Aihua Zhang

**Affiliations:** ^1^ Department of Nephrology, Nanjing Children's Hospital, Affiliated to Nanjing Medical University, Nanjing, China; ^2^ Jiangsu Key Laboratory of Pediatrics, Nanjing Medical University, Nanjing, China; ^3^ The First Clinical Medical College of Nanjing Medical University, Nanjing, China; ^4^ Department of Medicine, Mount Sinai School of Medicine, New York, NY, USA; ^5^ Division of Nephrology, Department of Medicine, Mount Sinai School of Medicine, New York, NY, USA

**Keywords:** albuminuria, NLRP3 inflammasome, COX-2, mPGES-1, NKCC2

## Abstract

Renal resistance to loop diuretics is a frequent complication in a number of kidney disease patients with elusive mechanism. Employing human renal biopsy specimens, albumin overload mouse model, and primary cultures of mouse renal tubular cells, albuminuria effect on NKCC2 expression and function and the underlying mechanisms were investigated. In the renal biopsy specimens of albuminuric patients, we found that NKCC2 was significantly downregulated with a negative correlation with albuminuria severity as examined by immunohistochemistry. Meanwhile, NLRP3 and mPGES-1 were stimulated in NKCC2 positive tubules (thick ascending limb, TAL) paralleled with increased urinary PGE2 excretion. To examine the role of albuminuria in the downregulation of NKCC2 and the potential role of NLRP3/prostaglandin signaling in NKCC2 downregulation, an albumin overload mouse model was employed. Interestingly, we discovered that albuminuria downregulated NKCC2 protein expression in murine kidney and impaired the renal response to loop diuretic furosemide. Specifically, albuminuria suppressed NKCC2 expression and function through NLRP3/prostaglandin dependent signaling in TAL. In primary cultures of renal tubular cells, albumin directly reduced NKCC2 but enhanced NLRP3, COX-2, and mPGES-1 expression. These novel findings demonstrated that albuminuria is of importance in mediating the renal resistance to loop diuretics via NLRP3/prostaglandin signaling-dependent NKCC2 downregulation in TAL. This may also offer novel, effective targets for dealing with the resistance of loop diuretics in proteinuric renal diseases.

## INTRODUCTION

Disordered salt and water handling is a common complication of many types of kidney disease. Nephrotic syndrome (NS) often presents with salt and water retention that is resistant to loop diuretics (NKCC2 inhibitors, e.g., furosemide), through mechanisms which are uncertain. Over the past decades, though substantial progress has been made in understanding the physiologic role of kidneys in regulating salt and water balance, the pathogenesis of the renal resistance to loop diuretics in NS remains elusive. Recent data suggest that intra-renal autocrine/paracrine factors contribute to disordered salt and water metabolism observed in NS. Since albuminuria is a hallmark feature of NS, we hypothesize that albuminuria is one of the key elements contributing to the phenomenon of the renal resistance to loop diuretics in albuminuric renal diseases.

Albuminuria is not only a hallmark of kidney diseases but also an independent pathogenic factor promoting the progression of kidney injury [1–3]. Recent reports have demonstrated that albumin is a potent activator of the NOD-like receptor (NLR) family, pyrin domain containing 3 (NLRP3) inflammasome in renal tubular cells [4] and that activation of the NLRP3 inflammasome is positively correlated with proteinuria severity [5]. The inflammasome is an intracellular multi-protein complex comprising caspase-1, PYCARD and NALP that initiates the innate immune response to a pathogen-associated and/or damage-associated molecular pattern. This process can cause cellular injury through inflammatory cytokines (IL-1β and IL-18) activated by caspase-1 [6–8]. A number of danger signals, including oxidative stress, monosodium urate and potassium efflux can activate inflammasomes [6, 9, 10]. Inflammasomes include NLR and pyrin and HIN200 domain-containing protein (PYHIN) families. Among the NLR family members (NLRP1, NLRP2, NLRP3, NLRP6, NLRP12, and NLRC4), NLRP3 deficient mice had less tubular injury, inflammation, and renal fibrosis in chronic kidney disease (CKD) [11].

In addition, caspase-1 and IL-1β have been shown to stimulate cyclooxygenase-2 (COX-2) dependent prostaglandin E2 (PGE2) release in murine injury models [12] and renal medullary interstitial cells [13]. In the kidney, PGE2 inhibits Na and water transport to support diuresis [14–17] while inhibition of PGE2 synthetic enzymes (COX-2 and membrane associated PGE synthase-1 [mPGES-1]) enhances Na and water transport to generate a concentrated urine [18–20]. In the thick ascending limb (TAL), PGE2 suppresses the expression and/or activity of the Na-K-2Cl- (NKCC) cotransporter [21], the site of action of furosemide, while inhibition of COX activity stimulates the abundance of NKCC2 in TALs [22]. Furthermore, Jia et al. suggested that lithium-induced polyuria and impaired urinary concentrating capacity were related to the actionof mPGES-1-derived PGE2 in suppressing NKCC2 expression/activity [23], emphasizing the importance of intra-renal PGE2 release on tubular Na transporters.

In the present study, employing the albumin overload mouse model, human renal biopsy specimens, and primary renal tubular cells, we investigated: 1) whether albuminuria was a contributor of NKCC2 downregulation and dysfunction; 2) whether NLRP3/prostaglandin served as the mechanistic signaling pathway in mediating albuminuria effect on NKCC2 reduction; 3) whether the dysregulation of NLRP3/prostaglandin signaling led to the impaired renal response to loop diuretics under albuminuric condition.

## RESULTS

### Albuminuric patients have increased NLRP3 and mPGES-1/PGE2 signaling and altered NKCC2 expression

**Figure 1:**
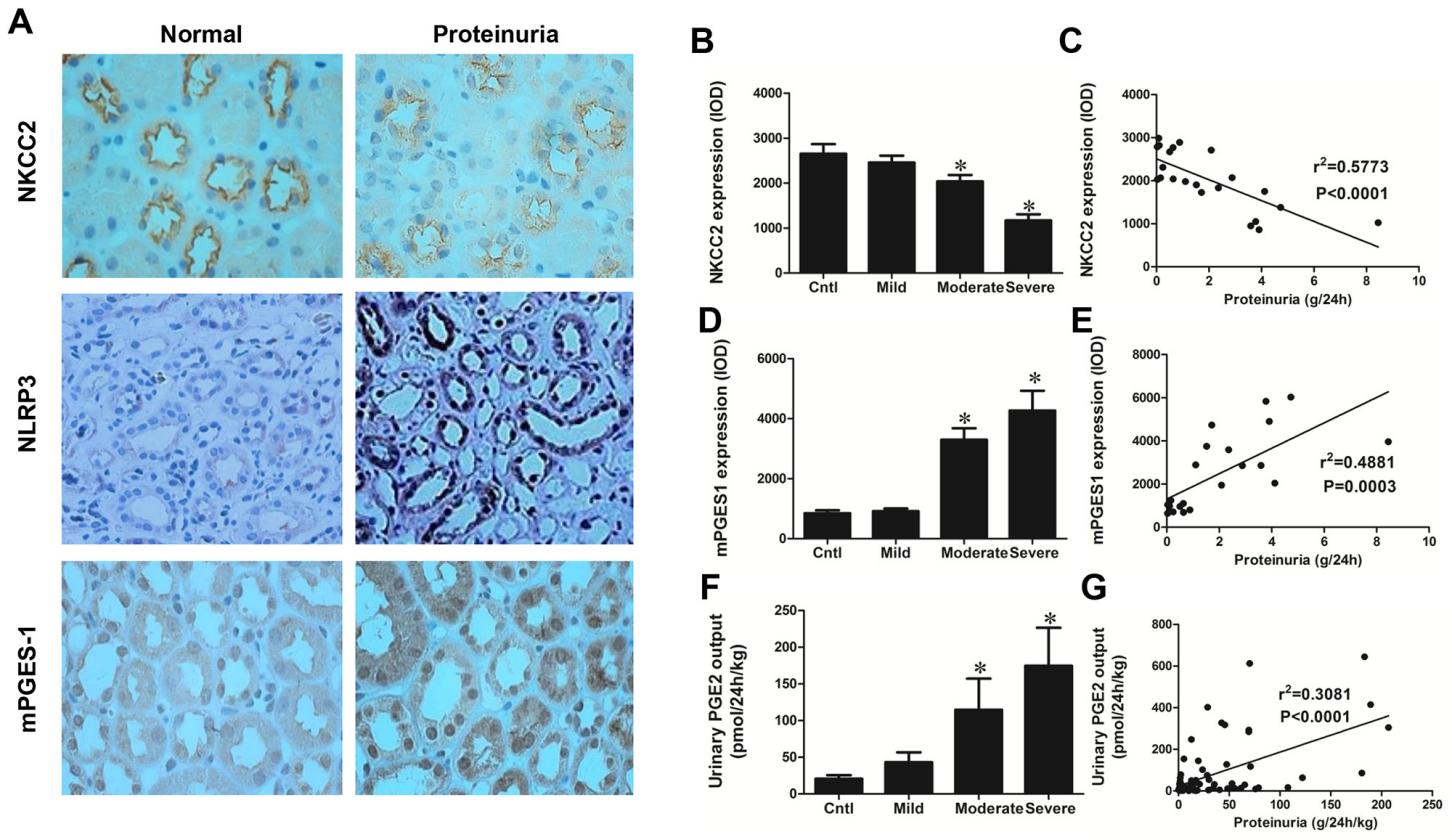
Analyses of NKCC2, NLRP3, mPGES-1, NKCC2, and PGE2 in the proteinuric patients **A.** Immunohistochemistry of NLRP3 and mPGES-1 illustrate their upregulation in albuminuric kidneys while NKCC2 expression is suppressed in the same kidneys. **B.** Quantification of NKCC2 expression in tubular epithelia decreases as proteinuria increases. **C.** Correlation analysis of NKCC2 with proteinuria identifies a negative correlation. **D.** Tubular expression of mPGES-1 increases with increasing proteinuria and correlation analysis **E.** of mPGES-1 vs. proteinuria demonstrates a positive correlation. **F.** Urinary PGE2 excretion, as determined by EIA, increases with proteinuria and this effect is confirmed by **G.** correlation analysis. The values represent the means±SDs. For urinary PGE2 assay, n=12-33 in each group. In other experiments, n=6 in each group. * p<0.01 vs. control group.

**Figure 2:**
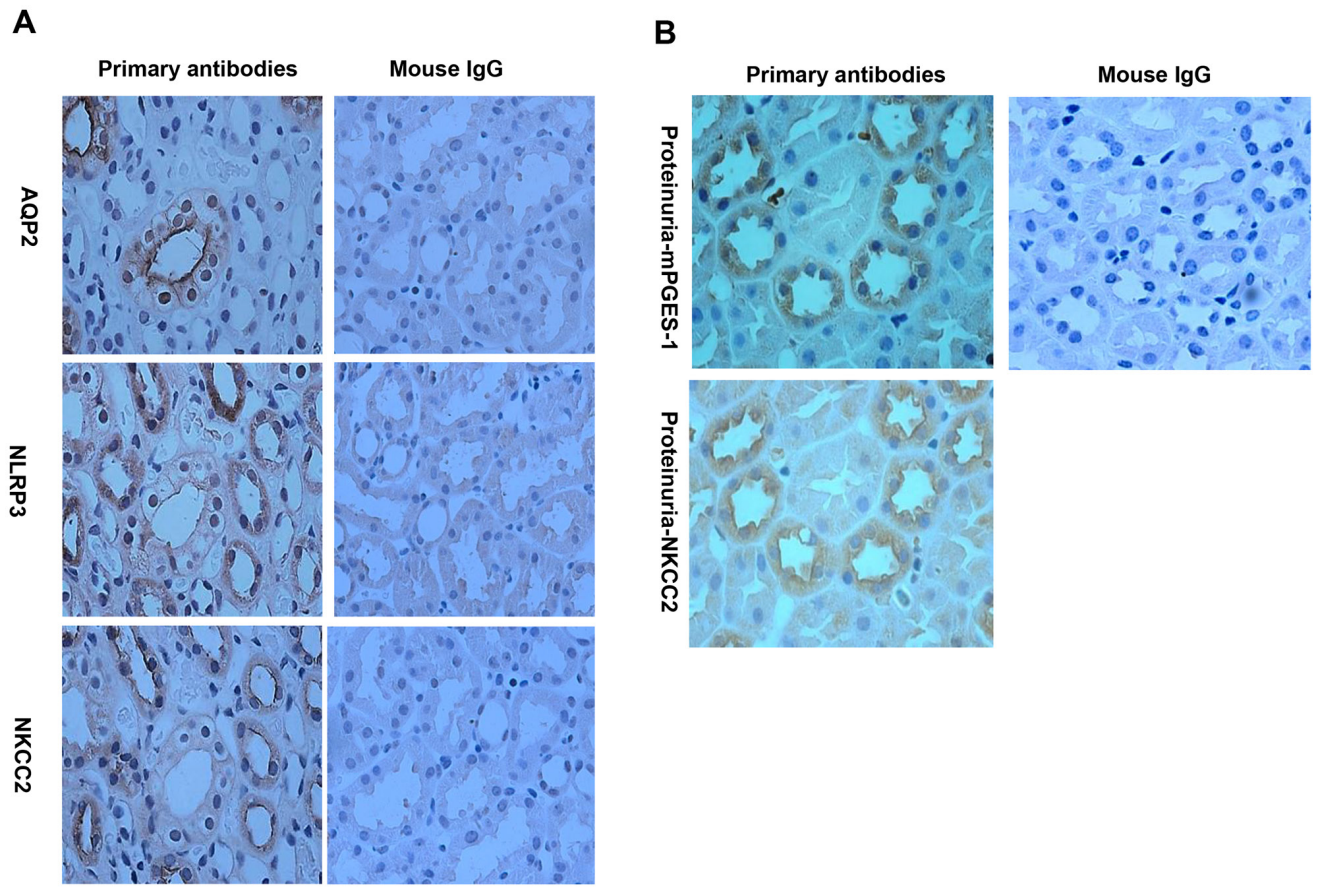
Localization analyses of NLRP3 and mPGES-1 in renal biopsy specimens of the proteinuric patients by immunohistochemistry **A.** NLRP3 is co-localized to tubular epithelial cells expressing NKCC2 by immunohistochemistry. **B.** mPGES-1 is also expressed in NKCC2 expressing tubular epithelial cells.

Kidney biopsy specimens from patients with primary glomerular disease who presented with obvious proteinuria without reaching the diagnostic standard of nephrotic syndrome (plasma albumin no less than 30 g/L) were chosen for the analysis to avoid the influence of blood volume depletion cause by fluid redistribution. As shown by the data, proteinuric patients showed remarkably elevated NLRP3 expression in TALs (NKCC2-positive tubules) (Figure 1A & 2A), in contrast with the markedly reduced NKCC2 expression, which negatively correlated with proteinuria (Figure 1A–1C). In line with the increase of NLRP3, mPGES-1 was also induced in TALs (Figure 1A, 1D and 2B), in parallel with urinary PGE2 production (Figure 1F). Both mPGES-1 and urinary PGE2 showed a positive correlation with proteinuria severity (Figure 1E & 1G).

### Albumin overload induced NLRP3 expression in the TAL

**Figure 3:**
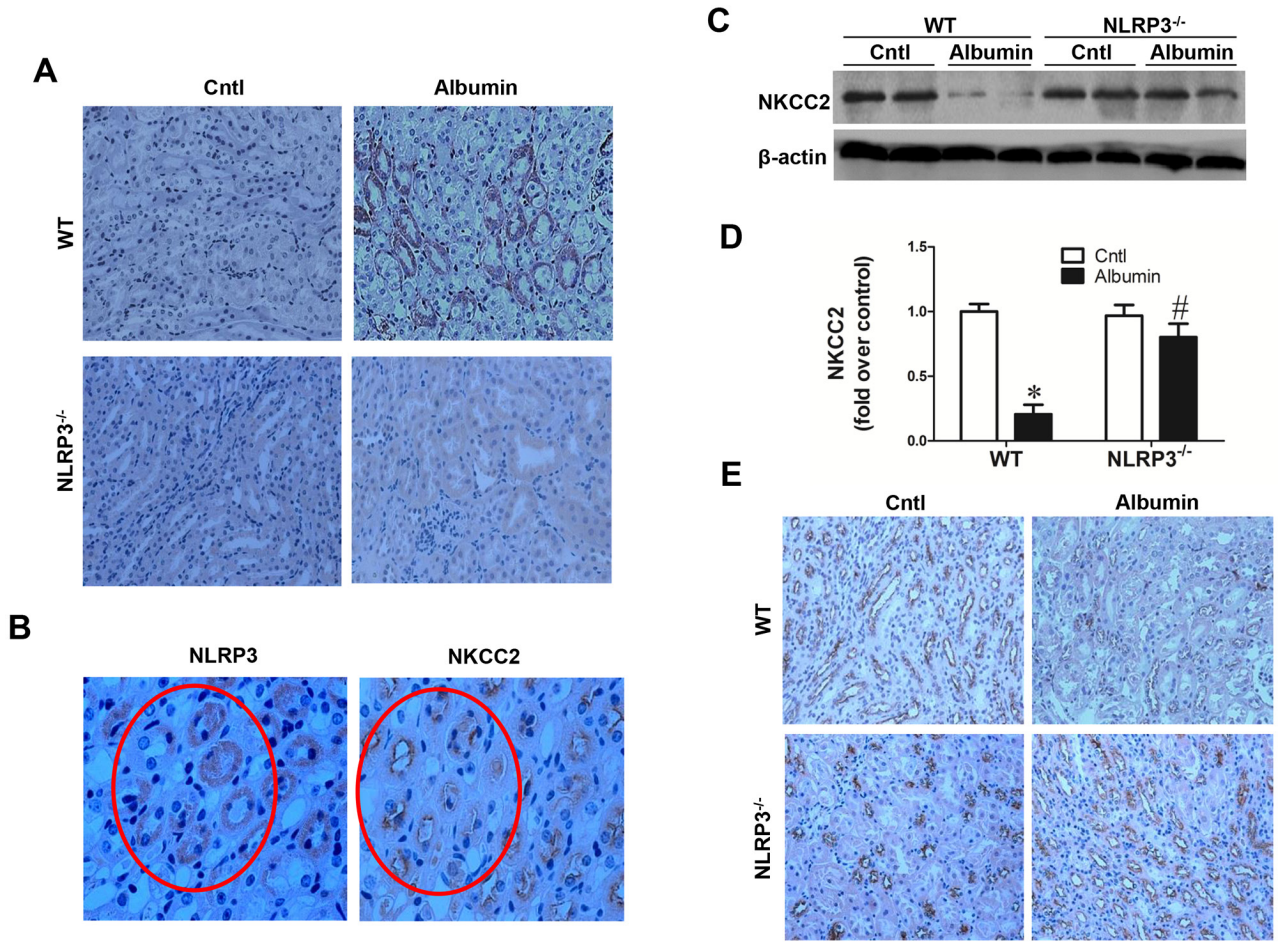
NLRP3 deletion reversed albuminuria-induced NKCC2 downregulation **A.** NLRP3 is induced in WT mice by albumin loading and is induced in the NKCC2 expression tubules **B. C.** Western blots of NKCC2. **D.** Densitometric analysis of NKCC2. **E.** Immunohistochemistry of NKCC2, qualitatively, demonstrates the NLRP3 dependence of albumin-induced NKCC2 expression. The values represent the means±SDs (n=6). * p<0.01 vs. WT or NLRP3^−/−^ control mice. # p<0.01 vs. albumin-overloaded WT mice.

Employing an albumin-overload mouse model, we observed a significant increase of NLRP3 expression in renal tubules of WT mice contrasting to an absence of NLRP3 signaling in NLRP3^−/−^ animals (Figure 3A). As shown by IHC of consecutive slides, NLRP3 was located at NKCC2-positive tubules, indicating an increase of NLRP3 in the TAL (Figure 3B). Mice with albumin loading showed comparable levels of blood albumin, Urea, and Creatinine as compared with the vehicle-treated controls (Table 1).

**Table 1:** Blood albumin, creatinine, and urea levels in WT mice

	Cntl	Albumin overload
Mouse albumin(g/l)	36.78(2.68)	32.14(2.04)
Creatinine(umol/l)	27.98(2.54)	22.47(2.71)
Urea(mmol/l)	12.86(1.46)	13.68(1.93)

### Effect of NLRP3 deletion on the alteration of NKCC2 induced by albumin overload

To investigate whether NLRP3 contribute to the alteration of NKCC2 under proteinuric condition, NLRP3 WT and NLRP3^−/−^ mice were treated with albumin overload. Interestingly, Western blotting analysis showed that NKCC2 protein levels were significantly reduced by 80% following albumin overload for 11 days in WT mice (Figure 3C&3D). Strikingly, NLRP3 deletion entirely abolished this reduction (Figure 3C&3D). By immunohistochemistry (IHC), we observed a similar regulation of NKCC2 as Western blotting results (Figure 3E).

### Activation of the COX-2/mPGES-1/PGE2 pathway in the kidney following the albumin overload was diminished by NLRP3 deletion

**Figure 4:**
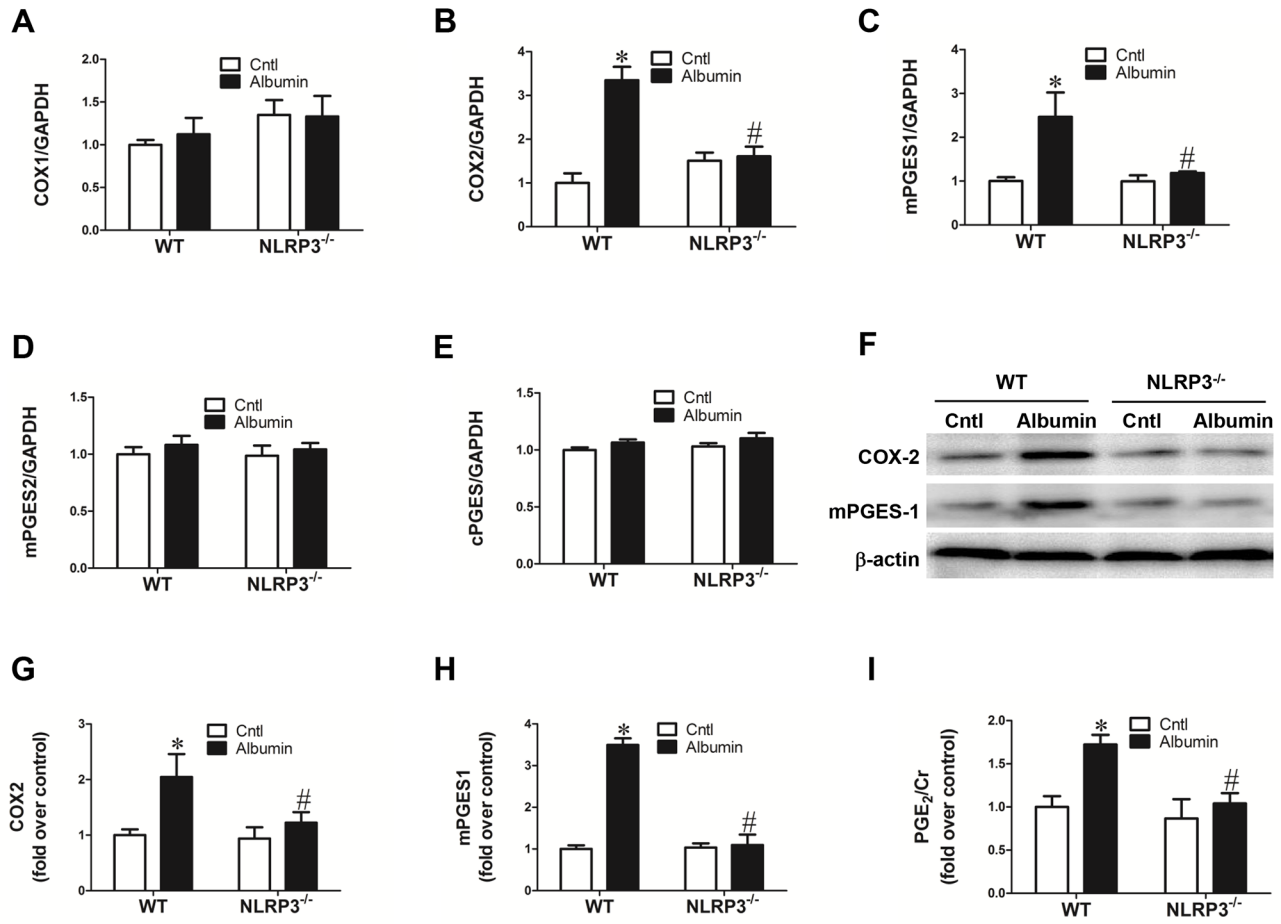
Activation of COX-2/mPGES-1/PGE2 pathway in the kidney following albumin overload was inhibited by NLRP3 deletion qRT-PCR analyses of COX-1 **A.** COX-2 **B.** mPGES-1 **C.** mPGES-2 **D.** and cPGES **E. F.** Western blots of COX-2 and mPGES-1, enzymes along the PGE2 synthetic pathway, are induced by albumin loading, but this effect is reversed in the NLRP3^−/−^ mice. **G–H.** Densitometric analyses of COX-2 (G) and mPGES-1(H) confirm the impression of the immunoblotting. **I.** EIA of urinary PGE2 excretion is similarly stimulated by albumin overloaded mice while reduced to near normal levels in NLRP3^−/−^ mice injected with albumin. The values represent the means±SDs (n=6 in each group). * p<0.01 vs. WT control. # p<0.01 vs. albumin-overloaded WT mice.

**Figure 5:**
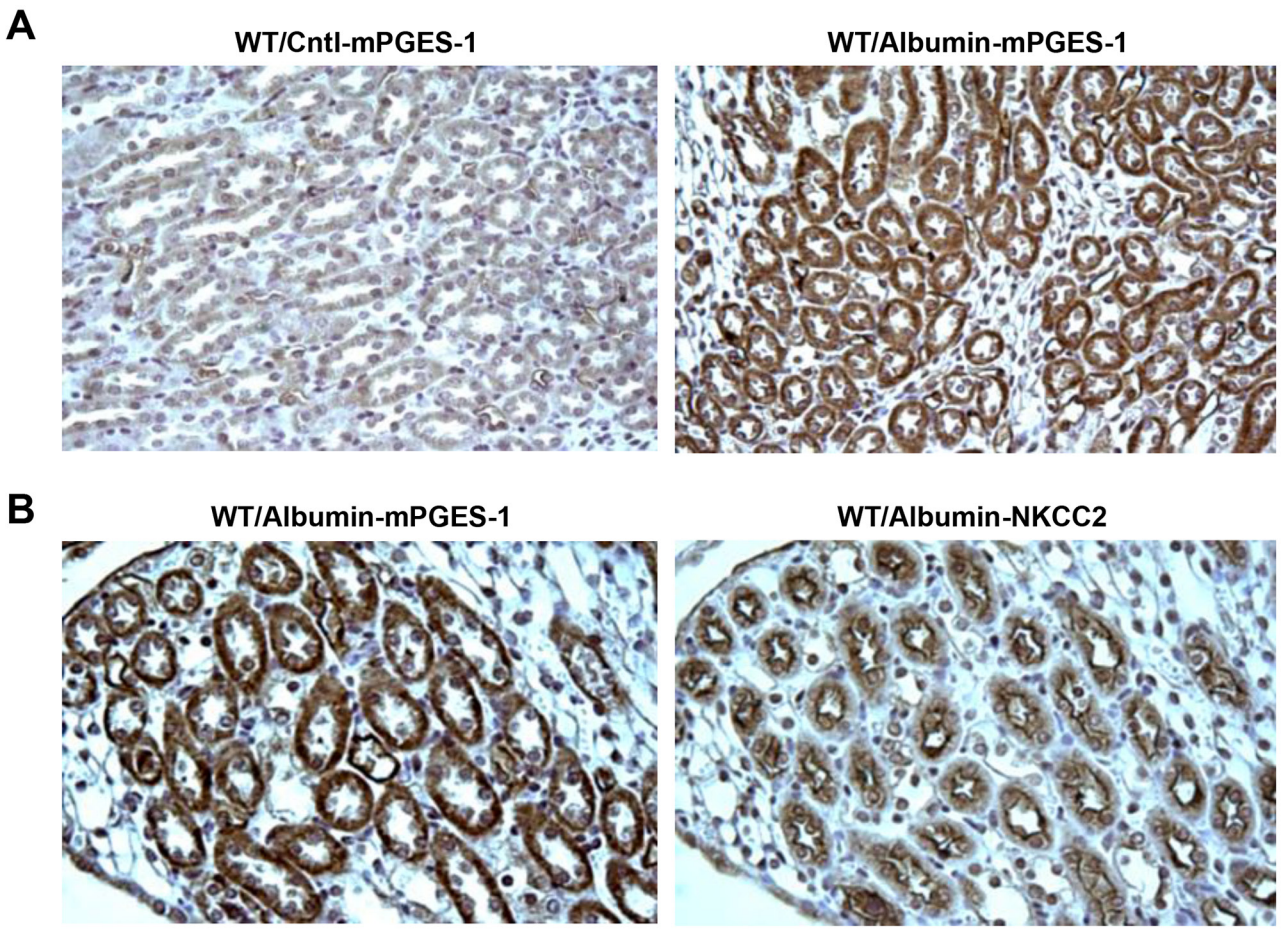
Localization of induced mPGES-1 in mouse kidney following albumin overload **A.** Immunohistochemistry of mPGES-1 illustrates the increase of mPGES-1 in tubules of albumin-loaded WT mice while consecutively stained section of kidney for albumin-loaded WT mice demonstrate **B.** mPGES-1 expression occurs specifically in the cells expressing NKCC2 (i.e. the TAL).

To further investigate the possible downstream signaling mediating the NLRP3 effect on NKCC2 regulation, we examined the COX-2/mPGES-1/PGE2 pathway in NLRP3 WT and NLRP3^−/−^ mice. As expected, the COX-2 (WT/Albumin 3.35 ± 0.31 vs. WT/Cntl 1.00 ± 0.22, *p*<0.01) and mPGES-1 (WT/Albumin 2.46 ± 0.56 vs. WT/Cntl 1.00 ± 0.09, *p*<0.01) mRNA expression was remarkably enhanced in WT mice following albumin overload (Figure 4B & 4C). In contrast, both albumin overload and NLRP3 deletion had no effect on the mRNA expression of COX-1, mPGES-2, or cPGES (Figure 4A, 4D & 4E). The qRT-PCR results for COX-2 and mPGES-1 expression were further confirmed by western blotting (Figure 4F-4H). As shown by ELISA, urinary PGE2 excretion was significantly elevated in WT mice (WT/Albumin 1.72 ± 0.11 vs. WT/Cntl 1.00 ± 0.13, *p*<0.05) after albumin overload (Figure 4I), and this effect was largely normalized by NLRP3 deletion (Figure 4I). Furthermore, IHC of consecutive slides showed that the increase of mPGES-1 also occurred in the TALs of the mice (Figure 5A&5B).

### Diuretic responses to furosemide in albumin-overloaded mice

**Figure 6:**
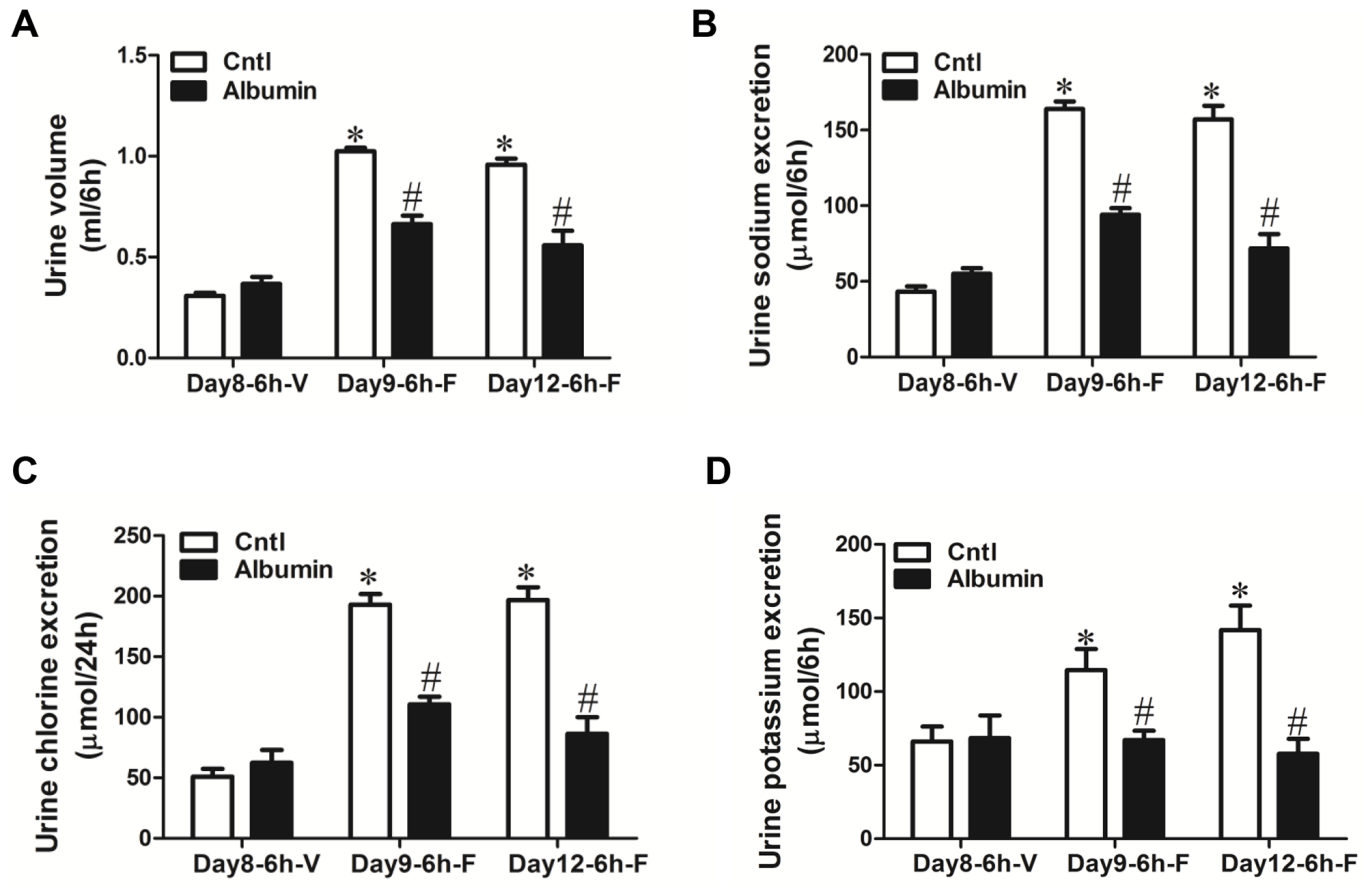
Physiologic study to test the diuretic response of albumin-overloaded WT mice to the NKCC2 inhibitor furosemide (F) **A–D.** WT mice were treated with either saline or albumin for up to 12 days and their diuretic response to vehicle **V.** or furosemide measured over 6 hr. Albumin overload reduced the urine volume (A) and urinary excretion of sodium (B), chloride (C), and potassium (D) in response to furosemide treatment. The values represent the means±SDs; n=6 in each group. *p<0.01 vs. vehicle-treated control mice. # p<0.01 vs. furosemide-treated control mice.

**Figure 7:**
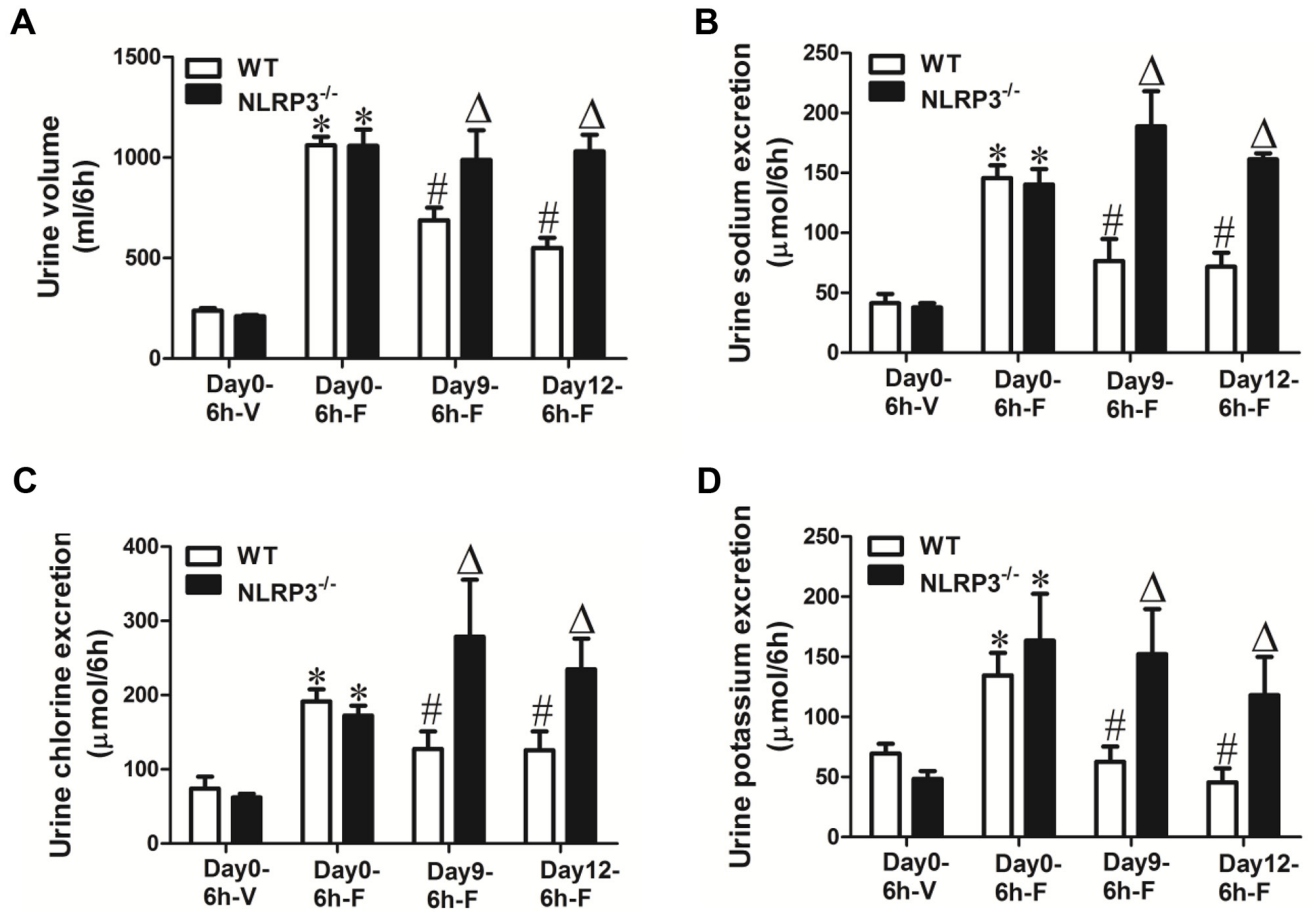
Physiologic study to test the diuretic response of albumin-overloaded WT and NLRP3^-/-^ mice to the NKCC2 inhibitor furosemide (F) **A–D.** WT and NLRP3^−/−^ mice were treated with either saline or albumin for up to 12 days and their diuretic response to vehicle (V) or furosemide measured over 6 hr. NLRP3 deletion reversed the blunted response to furosemide in albumin-overloaded mice. (A) Urine volume, (B) urinary sodium excretion, (C) urinary chloride excretion, and (D) urinary potassium excretion. The values represent the means±SDs (n=6 in each group). * p<0.01 vs. vehicle-treated WT or NLRP3^−/−^ mice without albumin overload (day0). # p<0.01 vs. furosemide-treated WT mice without albumin overload (day0). Δ p<0.01 vs. furosemide-treated WT mice following albumin overload for 9 days(day9) or 12 days (day12).

To further study the functional role of NKCC2 reduction in mice exposed to albumin overload, the loop diuretic furosemide (10 mg/kg body weight), an NKCC2 inhibitor, was administered to WT mice via i.p injection after albumin overload, and urine was collected for 6 h and analyzed. In line with the reduction of NKCC2 expression, the diuretic and natriuretic responses to furosemide were significantly blunted (Figure 6A–6D). In another experiment, furosemide was administered to WT and NLRP3^−/−^ mice before and after albumin overload. As shown by the data, before albumin overload, the response to furosemide was comparable between WT and NLRP3^−/−^ mice (Figure 7A–7D); however, after albumin overload, the impaired response to furosemide that was observed in WT mice was completely reversed in NLRP3^−/−^ mice (Figure 7A–7D).

### COX-2 inhibition in albumin-overloaded mice reversed NKCC2 downregulation and the diuretic resistance to furosemide

**Figure 8:**
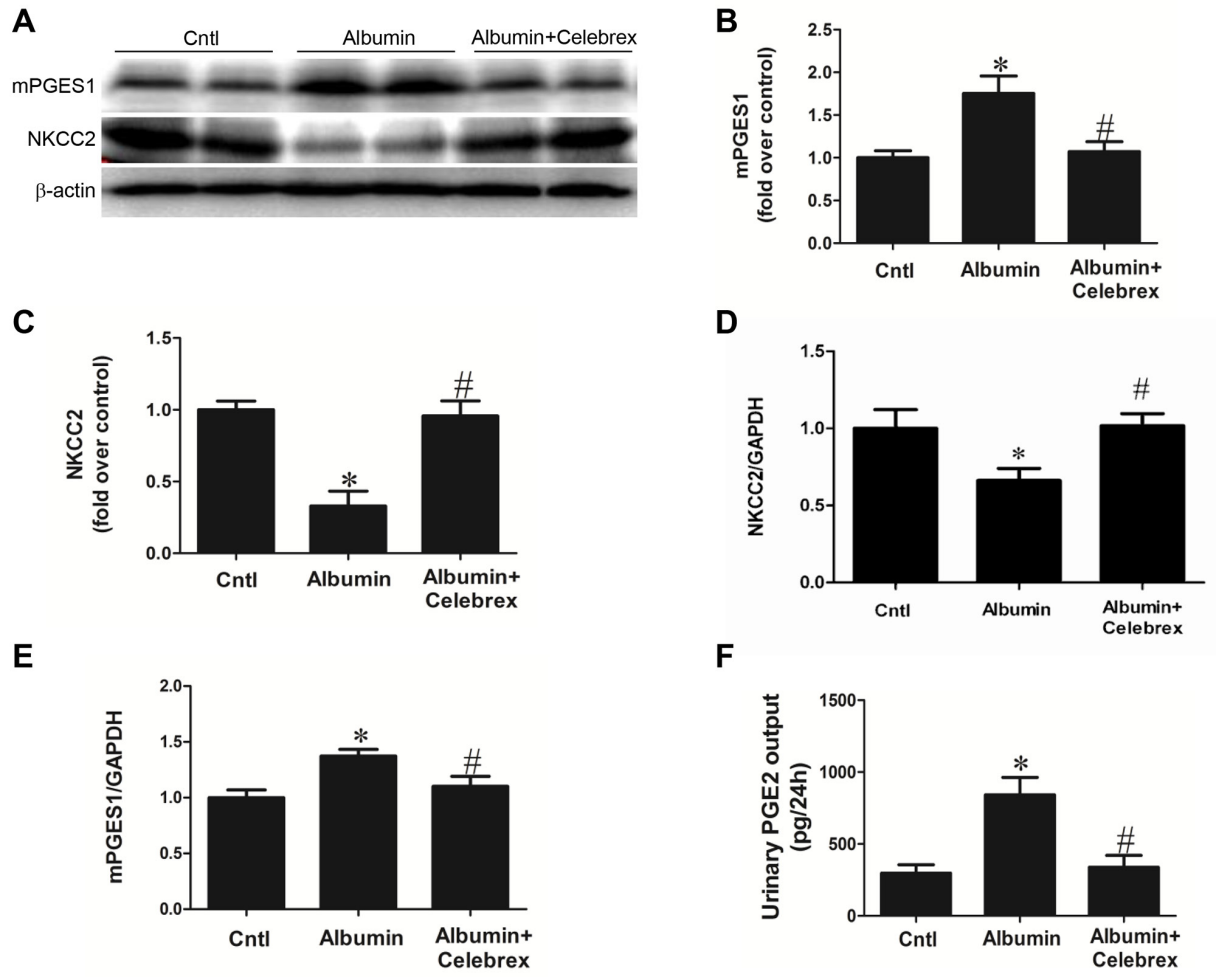
Celebrex treatment reversed the albuminuria-induced alteration of NKCC2 and involved signaling pathway **A.** Western blots of mPGES-1, NKCC2. **B & C.** Densitometric analyses of mPGES-1 (B) and NKCC2 (C). **D.** qRT-PCR analysis of NKCC2. **E.** qRT-PCR analysis of mPGES-1. **F.** EIA of urinary PGE2. n=6 in each group. * p<0.01 vs. control. # p<0.01 vs. albumin-overloaded mice.

**Figure 9:**
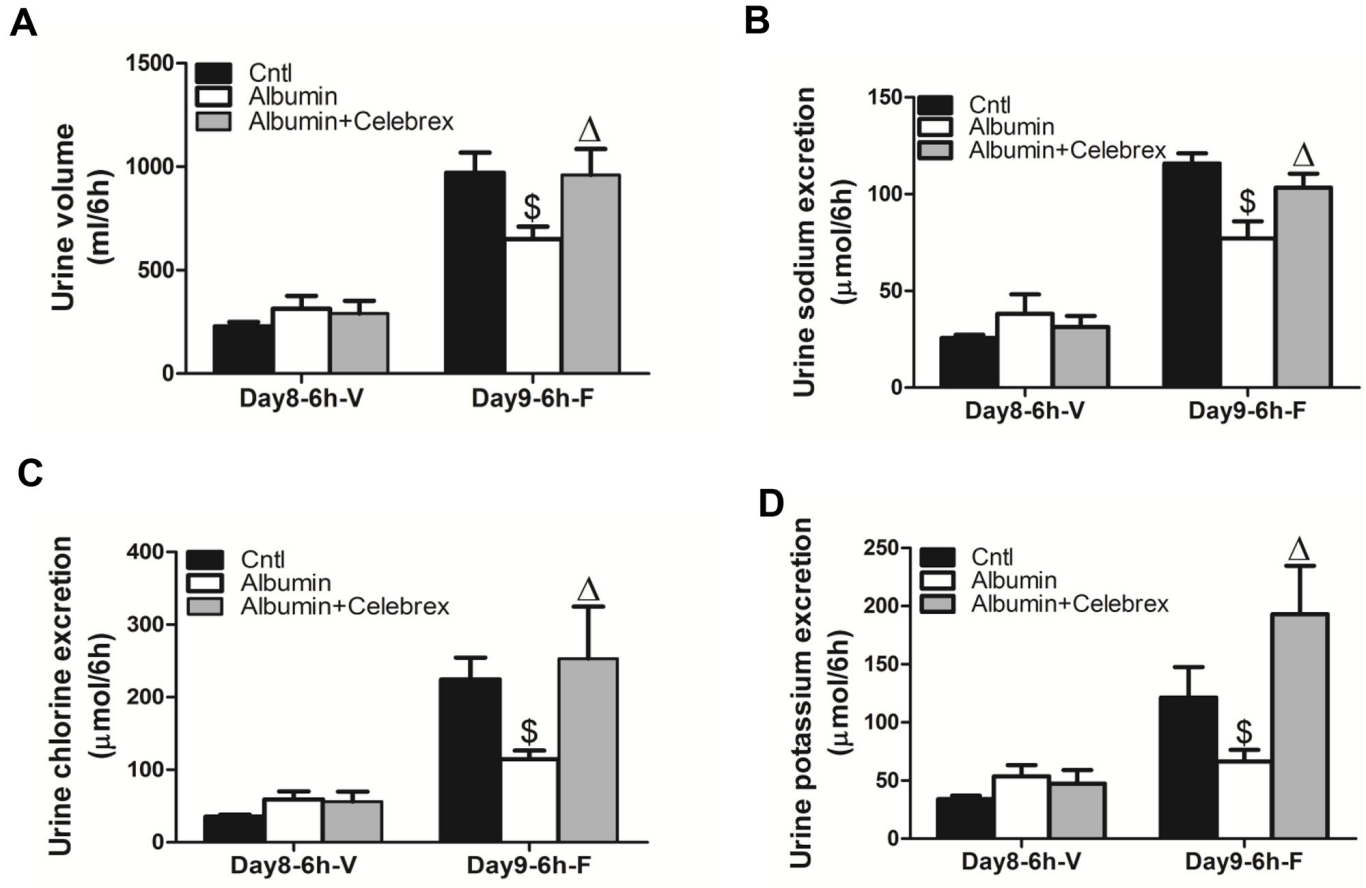
Celebrex treatment reversed the albuminuria-induced diuretic resistance to furosemide **A.** Urine volume, **B.** urinary sodium excretion, **C.** urinary chloride excretion, and **D.** urinary potassium excretion. The values represent the means±SDs (n=6 in each group). $ p<0.01 vs. furosemide-treated control mice (no albumin overload). Δ p<0.05 vs. furosemide-treated mice with albumin overload for 9 days (day9).

By applying the specific COX-2 inhibitor Celebrex (30 mg/kg/day in a jelly diet), the downregulation of NKCC2 protein (WT/Albumin+Celebrex 0.96 ± 0.09 vs. WT/Albumin 0.33 ± 0.10, *p*<0.01) and mRNA (WT/Albumin+Celebrex 1.02+0.08 vs. WT/Albumin 0.66 ± 0.08, *p*<0.01) expressions were completely restored (Figure 8A, 8C, and 8D), and the increase of urinary PGE2 was abolished in line with inhibition of mPGES-1 upregulation (Figure 8A, 8B, 8E, and 8F). In a separate experiment, in agreement with the restoration of NKCC2 by COX-2 inhibition, the impaired diuretic response to furosemide in albumin-treated WT mice was corrected by COX-2 inhibition (Figure 9A & 9D).

### Albumin effects on NKCC2 regulation and its involved signaling pathway in primary renal tubular cells

**Figure 10:**
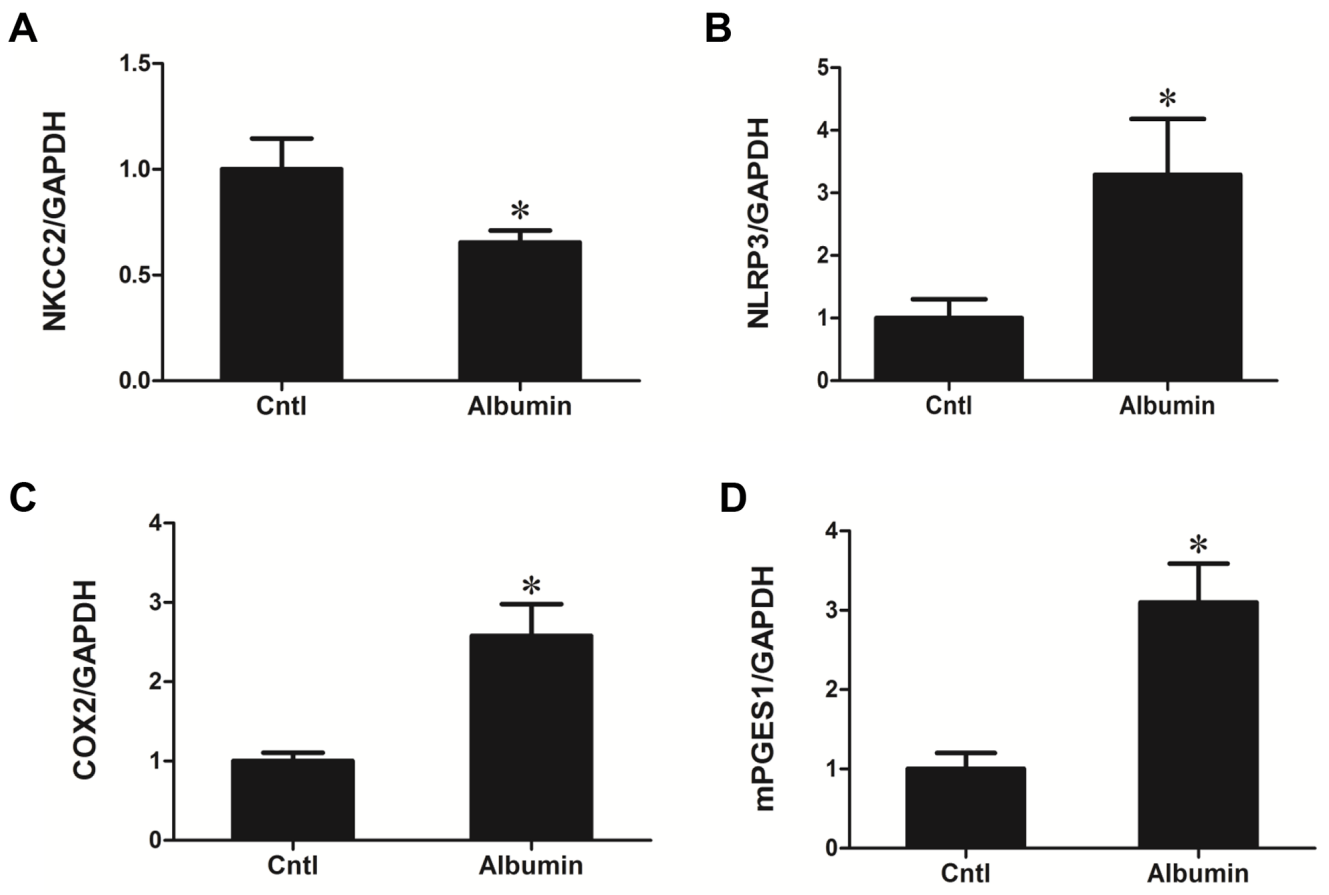
Albumin effects on the regulation of NKCC2 and involved signaling pathway in primary renal tubular cells qRT-PCR analysis of **A.** NKCC2, **B.** NLRP3, **C.** COX-2, and **D.** mPGES-1 mRNA expression following albumin treatment in primary renal tubular cells. The values represent the means±SDs (n=6 in each group). * p<0.01 vs. control.

As expected, albumin treatment significantly downregulated NKCC2 expression as determined by qRT-PCR (Albumin 0.66 ± 0.06 vs. Cntl 1.00 ± 0.14, *p*<0.01) (Figure 10A), indicating a direct effect of albumin on NKCC2 regulation. Meantime, albumin enhanced NLRP3 (Albumin 3.29 ± 0.89 vs. Cntl 1.00 ± 0.31, *p*<0.01), COX-2 (Albumin 2.58 ± 0.39 vs. Cntl 1.00 ± 0.10, *p*<0.01), and mPGES-1 (Albumin 3.10 ± 0.49 vs. Cntl 1.00 ± 0.20, *p*<0.01) mRNA expression in the primary renal tubular cells (Figure 10B&10C).

## DISCUSSION

**Figure 11:**
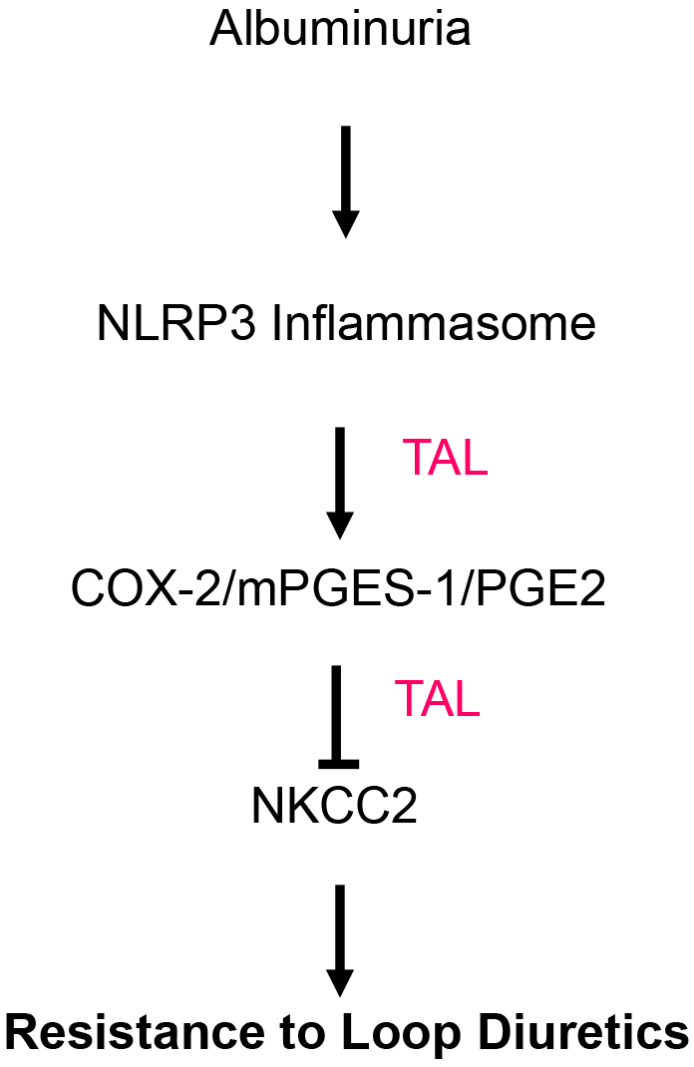
Schematic illustration of the hypothesis Albuminuria suppresses NKCC2 via the NLRP3 inflammasome/PGE2 axis in the TAL.

Abnormalities of Na and water metabolism are common clinical features of kidney disease that lead to fluid retention, edema, hypertension, and congestive heart failure. The diuretics, particularly the loop diuretics are of vital importance in dealing with the kidney disease-related fluid retention. However, a known phenomenon called resistance to loop diuretics in kidney disease patients, particularly in those NS patients markedly limited the efficacy of loop diuretics in diuresis with uncertain mechanisms. The major finding of this paper is that albuminuria stimulates the NLRP3 inflammasome to activate intra-renal COX-2/mPGES-1/PGE2 signaling that leads to suppression of NKCC2 (Figure 11). These findings were corroborated utilizing several methods (molecular and physiologic) and models (primary renal epithelial cell culture, murine kidney, and human kidney) to strengthen their validity.

In the present study, NKCC2 was downregulated in albumin overload mouse model, albumin-treated primary renal tubular cells, and kidneys of albuminuric patients. A lack of NKCC2 could be a contributing factor to the renal resistance to loop diuretics in albuminuric kidney diseases. Albumin binding to loop diuretics in the tubule fluid is considered a mechanism [24–27] for diuretic resistance, but it may not be the only mechanism. Agarwal et al. demonstrated that displacement of furosemide from its albumin binding site with sulfisoxazole did not restore the diuretic response to furosemide in patients with NS [28], implying that alternative mechanisms for diuretic resistance. We speculate that albumin-mediated suppression of NKCC2 expression may be another alternative mechanism of diuretic resistance.

In the kidney, PGE2 has an established role in inhibiting Na and water transport to cause diuresis [14–17]. In the TAL, COX-2 and PGE2 have been shown to suppress the expression and/or activity of the NKCC2 [21]. Moreover, inhibition of COXs in albuminuric renal disease is known to enhance tubular Na reabsorption which leads to edema and hypertension. This effect may, in part, be mediated by the upregulation of NKCC2 via suppressing PGE2 production. In agreement with these known concepts and findings, we observed a remarkable stimulation of COX-2/mPGES-1/PGE2 cascade in the kidneys of both albumin overloaded mice and albuminuric patients, which was negatively correlated with the downregulation of NKCC2. More interestingly, increased mPGES-1, a specific PGE2 synthase, was localized at TAL in both albumin overloaded animals and albuminuric patients. In line with the downregulation of NKCC2 in albumin overloaded mice, the diuretic response to furosemide, a most widely used loop diuretic, was significantly attenuated. Importantly, inhibition of COX-2 via a specific COX-2 inhibitor entirely normalized the impaired diuretic response to furosemide in parallel with the reversed NKCC2 downregulation. Meanwhile, the increased urinary PGE2 output was also abolished. All these novel findings demonstrated that albuminuria stimulated COX-2/mPGES-1/PGE2 cascade in TAL, which subsequently suppressed NKCC2 expression and led to the diuretic resistance to loop diuretics.

Next, what was the potential upstream signaling in mediating the albuminuria effect on stimulating COX-2/mPGES-1/PGE2 cascade in TAL? Considering our previous finding that NLRP3 inflammasome was activated in both animal kidneys following albumin overload and the biopsy renal specimens of patients with albuminuria, possibly through the mitochondrial oxidative stress [5], we hypothesized that NLRP3 inflammasome might serve as an upstream activator of COX-2/mPGES-1/PGE2 cascade in TAL. In agreement with this hypothesis, we found a stimulation of NLRP3 protein in NKCC2 positive tubules (TAL) in both albumin overloaded mice and albuminuric patients. More importantly, deletion of NLRP3 not only reversed NKCC2 downregulation and the resistance to furosemide but also entirely blocked the stimulation of COX-2/mPGES-1/PGE2 cascade. These findings highly suggested that NLRP3 inflammasome served as an upstream signaling component in stimulating COX-2/mPGES-1/PGE2 cascade which finally suppressed the NKCC2 expression, leading to the resistance to loop diuretics.

The strengths of our study lie in the diverse array of corroborating data generated using various methods and models; however, as with all studies, limitations exist. In particular, the murine model of nephrosis studied does not have podocyte injury and no generalized proteinuria, as is normally observed with NS. Though limiting because no human renal disease presents as such, this unique model permitted us to clearly study the effects of urinary albumin on renal transporter expression. In summary, our studies validated in mouse and in human, demonstrate the key role that albuminuria plays in effectuating changes of renal NKCC2 expression which contribute to the diuretic resistance to loop diuretics. The research findings not only increased our understanding of renal resistance to loop diuretics in kidney disease patients but also offered potential targets for the effective handling of this common phenomenon in the clinic.

## MATERIALS AND METHODS

### Animals

NLRP3-knockout (NLRP3^−/−^) mice raised in the C57BL/6J-129 background (The Jackson Laboratory, Sacramento, CA) were used in the present study. In particular, heterozygous littermates were bred to generate homozygous knockout mice and wild-type (WT) littermate controls. Genotypes were identified by PCR. All mice were maintained on a 12-hr light-dark cycle in a temperature-controlled (19-21°C) room, fed a standard rodent diet and allowed free access to drinking water. All procedures were in accordance with the guidelines approved by the Institutional Animal Care and Use Committee at Nanjing Medical University (No. 20090053).

### Reagents and antibodies

DMEM-F12, newborn bovine serum, HEPES and penicillin/streptomycin were purchased from Wisent Corporation (Wisent, Canada). Hank's balanced salt solution (HBSS), nonessential amino acids, sodium pyruvate, insulin-transferrin-selenium and L-glutamine were purchased from Invitrogen Life Technologies (Paisley, Scotland). Stainless steel sieves were obtained from Merck Eurolab (Leuven, Belgium). Anti-NKCC2 (Stressmarq Biosciences Inc., Canada), anti-NLRP3 (Abcam, Cambridge, MA), anti-mPGES1 and anti-COX2 (Cayman, Ann Arbor, MI) primary antibodies and horseradish peroxidase (HRP)-conjugated secondary antibodies (Santa Cruz Biotechnology, Santa Cruz, CA) were used.

### Human renal biopsy specimens

Renal biopsy samples were obtained from patients undergoing diagnostic evaluation at the Department of Nephrology of Nanjing Children's Hospital, which is affiliated with Nanjing Medical University. Eighteen subjects (age range: 2-15 years old) were selected based on the criterion of having at least ten glomeruli in a paraffin-embedded tissue sample available for histological sectioning. All biopsy specimens were evaluated by a pathologist who was unaware of the results of the molecular studies. The samples were divided into the following categories according to the severity of proteinuria: mild proteinuria (<1.0 g/24 h, n=6), moderate proteinuria (1.0-3.0 g/24 h, n=6), and severe proteinuria (>3.0 g/24 h, n=6). Normal renal tissues were obtained from patients without proteinuria who underwent partial nephrectomy for benign renal tumors. The study was approved by the ethics committee at Nanjing Children's Hospital, China (LL20130432).

### Human urine samples

Twenty-four-hour urine samples were collected from the patients (age range: 2-15 years old) after being hospitalized at the Department of Nephrology of Nanjing Children's Hospital. Fifty-six urine samples were collected from patients diagnosed with primary proteinuric kidney diseases but with serum albumin levels over 30g/L (Table 2). These samples were divided into the following categories according to the severity of proteinuria: mild proteinuria (<1.0 g/24 h, n=26), moderate proteinuria (1.0-3.0 g/24 h, n=12), and severe proteinuria (>3.0 g/24 h, n=18). Twenty-four-hour normal urine was also obtained from 33 hospitalized patients without proteinuria. Before urine was collected, we obtained permission from the parents of the patients. The urine was promptly centrifuged to remove the sediment and stored at -80°C. This study was approved by the ethics committee at Nanjing Children's Hospital, China (201407005-1).

**Table 2:** Blood albumin, creatinine, and urea levels in normal controls and albuminuric patients

	Cntl	Mild	Moderate	Severe
Albumin(g/l)	44.23(3.19)	41.47(4.05)	39.18(4.96)*	33.49 (5.98)*
Creatinine(umol/l)	41.07(12.09)	39.67(16.15)	44.59(14.04)	57.07(22.98)
Urea(mmol/l)	4.58(1.98)	4.48(2.01)	5.09(2.41)	5.16(2.39)

Mean (SD): *P<0.05 vs Cntl.

### Albumin overload experiments

Eight-week-old male mice (25-30 g each) received intraperitoneal (IP) injections daily for 11 days with low-endotoxin bovine serum albumin (BSA) (A-9430, Sigma Chemical Co., St. Louis, MO) dissolved in saline. BSA was administered for 5 days using a stepwise, incremental dose regimen, with the doses rising from 2 mg/g body weight on the first day (D1) to a maximum dose of 10 mg/g on the fifth day, which was maintained thereafter for 6 days.

In another experiment, WT and NLRP3^−/−^ mice were divided into four groups (n=6 per group): WT+vehicle, WT+albumin overload, NLRP3^−/−^+vehicle and NLRP3^−/−^+ albumin overload. As described above, the mice were subjected to daily IP injections of BSA or the same volume of saline for 14 days. In the Celebrex experiment (n=6 per group), the control mice subjected to daily IP injections of saline received a normal diet for 11 days, and the albumin-treated mice received control diet or a diet containing Celebrex (30 mg/kg/day in diet) for the same number of days. At the termination of the experiments, the mice were anesthetized with an IP injection of a ketamine/xylazine/atropine, and plasma and kidney samples were immediately frozen in liquid nitrogen and stored at -80°C until use.

### Responses to the loop diuretic furosemide

A 2.5 mg/ml furosemide (Sigma, St. Louis, MO) solution was prepared in 10% DMSO in deionized water at pH 7; 10% DMSO solution alone was used for the control injections. Mice were administered an intraperitoneal injection of 10 mg/kg body weight of the furosemide solution or vehicle control and were immediately placed into individual metabolic cages and provided free access to tap water. Urine was collected for exactly 6 hours, the urine volume was then measured, and the urine sodium, potassium and chloride concentrations were analyzed using a flame photometer (Instrumentation Laboratory, Lexington, MA).

### Primary culture of mouse renal tubular cells

Eight-week-old male mice were anesthetized with an IP injection of a ketamine/xylazine/atropine solution, their aortas were clamped off below the kidneys, and their kidneys were washed with phosphate-buffered saline (PBS). Following complete blood washout, the kidneys were excised, and the renal cortices manually dissected in ice-cold dissection solution (DS) (HBSS with 10 mmol/l glucose, 5 mmol/l glycine, 1 mmol/l alanine, and 15 mmol/l HEPES pH 7.4 at an osmolality of 325 mosmol/kg H_2_O) into 1-mm-wide pieces. The fragments were ground and sieved through two stainless steel sieves (pore sizes 80 microns and 160 microns) in DS, and the tubule fragments obtained from the 160-micron sieve were centrifuged for 5 min at 1000 r/min. After centrifugation, the supernatant was discarded, and 0.05% trypsin with EDTA) added for 20 min at 37°C to digest the tubular fragments. DMEM-F12 containing 10% FBS was used to stop the digestion. The solution containing the tubular fragments was then centrifuged for 5 min at 1000 r/min, and after discarding the supernatant, the tubular cells were resuspended in the appropriate amount of culture medium (DMEM/F12 supplemented with 10% newborn bovine serum, 15 mmol/l HEPES, 2 mmol/l L-glutamine, 50 nmol/l hydrocortisone, 5 g/ml insulin, 5 g/ml transferrin, 50 nmol/l selenium, 0.55 mmol/l sodium pyruvate, 10 ml/l 100x nonessential amino acids, 100 IU/ml penicillin and 100 g/ml streptomycin buffered to pH 7.4 at an osmolality of 325 mosmol/kg H_2_O). The tubular cells were then seeded onto collagen-coated 6-well cell culture plates (Greiner, Germany) and left unstirred for 48 h at 37°C and 95% air-5% CO_2_ in a standard humidified incubator (Thermo, UK), after which the culture medium was changed for the first time. The medium was then replaced every 2 days, and after 5 days, the cells grew into a confluent monolayer. Then, the cells were treated with albumin (10 mg/ml) for 24 h.

### Quantitative real-time PCR (qRT-PCR)

Total DNA and RNA were extracted using the DNeasy Tissue Kit (Qiagen, Valencia, CA, USA) and TRIzol reagent (Invitrogen), respectively. Oligonucleotides were designed using Primer3 software (available at http://frodo.wi.mit.edu/) and synthesized by Invitrogen. The sequences of the primer pairs are shown in Table 3. qRT-PCR was then used to detect the mRNA expression of the target genes, and reverse transcription was performed using a reaction kit (Promega Reverse Transcription System) according to the manufacturer's protocol. Real-time PCR amplification was performed using the ABI 7500 real-time PCR detection system (Foster City, CA, USA) with the SYBR Green PCR Master Mix (Applied Biosystems). The cycling conditions were 95°C for 10 min, followed by 40 cycles of 95°C for 15 s and 60°C for 1 min. The mRNA levels were normalized to a GAPDH control and calculated using the comparative cycle threshold (ΔΔCt) method.

**Table 3:** Primer sequences for qRT-PCR

Gene symbol	Primer sequences
GAPDH	5’-GTCTTCACTACCATGGAGAAGG-3’
	5’-TCATGGATGACCTTGGCCAG-3’
NKCC2	5’-GCTCTTCATTCGCCTCTCCT-3’
	5’-AGCCTATTGACCCACCGAAC-3’
mPGES-1	5’-GGATGCGCTGAAACGTGGA-3’
	5’-CAGGAATGAGTACACGAAGCC-3’
mPGES-2	5’-CCTCGACTTCCACTCCCTG-3’
	5’-TGAGGGCACTAATGATGACAGAG-3’
cPGES	5’-TGTTTGCGAAAAGGAGAATCCG-3’
	5’-CCATGTGATCCATCATCTCAGAG-3’
COX-1	5’-ATGAGTCGAAGGAGTCTCTCG-3’
	5’-GCACGGATAGTAACAACAGGGA-3’
COX-2	5’-AACCGTGGGGAATGTATGAG-3’
	5’-GCAGGAAGGGGATGTTGTT-3’
NLRP3	5’-GTGGTGACCCTCTGTGAGGT-3’
	5’-TCTTCCTGGAGCGCTTCTAA-3’

### Western blotting

mPTCs were lysed using a protein lysis buffer containing 50 mM Tris, 150 mM NaCl, 10 mM EDTA, 1% Triton X-100, 200 mM sodium fluoride, and 4 mM sodium orthovanadate, as a protease inhibitor (pH 7.5). Immunoblotting was then performed using primary antibodies against NLRP3 (1:500), NKCC2 (1:1000), mPGES1 (1:300), COX2 (1:500), and β-actin (1:1000), followed by the addition of HRP-labeled secondary antibodies. The blots were then visualized using the Amersham ECL detection system (Amersham, Little Chalfont, UK), and densitometric analysis was performed using Quantity One software (Bio-Rad).

### Immunostaining

Kidneys were fixed in 4% paraformaldehyde, embedded in paraffin and then cut into 3-µm-thick sections (Cryostat 2800 Frigocut-E, Leica Instruments), and a standard protocol using xylene and graded ethanol was employed to deparaffinize and rehydrate the tissues. The sections were washed with PBS and treated with blocking buffer containing 50 mM NH_4_Cl, 2% BSA, and 0.05% saponin in PBS for 20 min at room temperature. The sections were then incubated overnight at 4°C with primary antibodies of anti-NLRP3 rabbit polyclonal antibody (1:150), anti-NKCC2 rabbit polyclonal antibody (1:150), anti-mPGES1 rabbit polyclonal antibody (1:125). After washing with PBS, the secondary antibody was applied, and the signals were visualized using an ABC kit (Santa Cruz Biotechnology, Santa Cruz, CA).

### EIA assay

Urine samples were centrifuged for 5 min at 10,000 rpm and diluted 1:1 with enzyme immunoassay (EIA) Buffer. The PGE2 concentrations were then measured by EIA according to the manufacturer's instructions (Cayman, Ann Arbor, MI).

### Statistical analysis

All data are presented as the means ± standard deviations (SDs). Statistical analysis was performed using ANOVA followed by Bonferroni's test or unpaired Student's t test with the SPSS 13 statistical software. P<0.05 was considered significant.

## Abbreviations

NS: Nephrotic syndrome
NLRP3: NOD-like receptor family, pyrin domain containing 3
NLR: NOD-like receptor
PYHIN: pyrin and HIN200 domain-containing protein
CKD: chronic kidney disease
COX-2: cyclooxygenase-2
PGE2: prostaglandin E2
mPGES-1: membrane associated PGE synthase-1
TAL: thick ascending limb
NKCC: Na-K-Cl cotransporter
WT: wild-type
BSA: bovine serum albumin
IHC: immunohistochemistry.
